# Improving access to psychological therapy: Initial evaluation of two UK demonstration sites

**DOI:** 10.1016/j.brat.2009.07.010

**Published:** 2009-11

**Authors:** David M. Clark, Richard Layard, Rachel Smithies, David A. Richards, Rupert Suckling, Benjamin Wright

**Affiliations:** a NIHR Biomedical Research Centre for Mental Health, South London & Maudsley NHS Foundation Trust & Kings College London, UK; b London School of Economics, London, UK; c University of Exeter, UK; d Doncaster Primary Care Trust, UK; e East London NHS Foundation Trust, London, UK

**Keywords:** Dissemination, CBT, IAPT, Outcome monitoring, Routine services, Depression, Anxiety disorders

## Abstract

Recently the UK Government announced an unprecedented, large-scale initiative for Improving Access to Psychological Therapies (IAPT) for depression and anxiety disorders. Prior to this development, the Department of Health established two pilot projects that aimed to collect valuable information to inform the national roll-out. Doncaster and Newham received additional funds to rapidly increase the availability of CBT-related interventions and to deploy them in new clinical services, operating on stepped-care principles, when appropriate. This article reports an evaluation of the new services (termed ‘demonstration sites’) during their first thirteen months of operation. A session-by-session outcome monitoring system achieved unusually high levels of pre to post-treatment data completeness. Large numbers of patients were treated, with low-intensity interventions (such as guided self-help) being particularly helpful for achieving high throughput. Clinical outcomes were broadly in line with expectation. 55–56% of patients who had attended at least twice (including the assessment interview) were classified as recovered when they left the services and 5% had improved their employment status. Treatment gains were largely maintained at 10 month follow-up. Opening the services to self-referral appeared to facilitate access for some groups that tend to be underrepresented in general practice referrals. Outcomes were comparable for the different ethnic groups who access the services. Issues for the further development of IAPT are discussed.

## Introduction

On World Mental Health Day in October 2007 the UK Government announced an unprecedented, large-scale initiative for Improving Access to Psychological Therapies (IAPT) for depression and anxiety disorders within the English National Health Service. Between 2008 and 2011 investment in psychological therapies for these conditions will steadily rise to £173 million per annum above existing expenditure. The extra investment is being used to train and employ at least 3600 new psychological therapists who will work in new IAPT clinical services offering evidence-based psychological therapies that are recommended by the National Institute for Clinical Excellence (NICE). The training follows national curricula and initially particularly focuses on cognitive-behaviour therapy (CBT) as this is where the manpower shortage is considered greatest. Full details of the programme can be found in the IAPT Implementation Plan (Department of Health, 2008a) and associated documents (available from www.iapt.nhs.uk).

As with any large-scale health initiative, the IAPT programme has its roots in a wide range of clinical and policy developments. However, three developments deserve particular mention. First, between 2004 and 2007, NICE reviewed the evidence for the effectiveness of a variety of interventions and issued clinical guidelines (NICE., 2004a, 2004b, 2005a, 2005b, 2006) that strongly support the use of certain psychological therapies. CBT is recommended for depression and all the anxiety disorders. Some other therapies (interpersonal psychotherapy, couples therapy, counselling) are also recommended for depression, but not for anxiety disorders. In the light of evidence that some individuals respond well to “low-intensity” interventions (such as guided self-help and computerised CBT) NICE also advocated a stepped-care approach to the delivery of psychological therapies in mild to moderate depression and some anxiety disorders. In severe depression and some other anxiety disorders (such as post-traumatic stress disorder) low-intensity interventions are not recommended and instead it is suggested that patients should at once be offered “high-intensity” face-to-face psychological therapy.

In the second development, economists and clinical researchers combined resources to argue that an increase in access to psychological therapies would largely pay for itself by reducing other depression and anxiety-related public costs (welfare benefits and medical costs) and increasing revenues (taxes from return to work, increased productivity etc). This argument was advanced in the widely distributed *Depression Report* (Layard et al., 2006) and in academic articles (e.g. Layard, Clark, Knapp, & Mayraz, 2007).

In the third development, the Department of Health funded two pilot projects that aimed to collect valuable information to inform the national roll-out. Two of the 152 primary care trusts (PCTs) in England were selected and designated “demonstration sites”. These were Doncaster (in Yorkshire) and Newham (in inner London). Both received £1.3–1.5 million extra funding to develop expanded psychological treatment services for depression and anxiety disorders that primarily focused on delivering the CBT-related interventions recommended in NICE guidance, using a stepped-care approach for those conditions in which it was considered appropriate.

The Doncaster and Newham services opened in late summer 2006. In this article, which is an abbreviated version of a longer report (Clark, Layard, & Smithies, 2008) that is available on the IAPT website (www.iapt.nhs.uk), we document the achievements of the two sites up to end September 2007. This covers roughly the first year of operation, during which both sites were starting from scratch. A fuller evaluation based at the University of Sheffield and supported by the UK National Institute for Health Research Service Delivery and Organisation (SDO) will be following patients over a longer period. Further details of the Doncaster demonstration site can be also be found in Richards and Suckling (2008, 2009).

First, we describe the general features of the two demonstration sites and the methodology used in this evaluation. We then present the main findings from each site in turn before drawing some general conclusions.

## General features of the two demonstration sites

### Clinical populations

Both sites focused on individuals with depression and/or anxiety disorders. However, they concentrated on somewhat different populations. Doncaster focused predominantly on individuals for whom depression was considered by their general practitioner (GP) to be their main problem, although many were also considered to have generalized anxiety disorder. Post-traumatic stress disorder and obsessive-compulsive disorder were excluded. Newham focused on depression and all anxiety disorders. Individuals seen in Doncaster are predominantly white (in line with local demographics), whereas Newham has an ethnically mixed population with a significant number of people who do not usually speak English.

### Services

The clinical services in both sites are based on NICE Guidelines, but have somewhat different emphases. Doncaster is described as a high throughput, stepped-care service with a marked emphasis on low-intensity work (especially guided self-help), although high-intensity work is also available. Newham places a greater emphasis on high-intensity CBT but over time has increased its capacity to deliver low-intensity interventions for the conditions where they are indicated.

### Referrals

In order to widen access, multiple sources of referral were allowed. The largest group of patients were referred by their GPs, as is usual in the UK National Health Service (NHS). However, referrals were also accepted from employment support agencies (Job Centre Plus) and other health professions. In a break with usual NHS tradition, self-referral was also allowed as an experiment to determine whether it identified people who would not otherwise have access to services. Newham made extensive use of self-referral.

## Methods

### Design

An observational, prospective cohort study. All patients who were assessed by the services were asked to complete standardized measures of depression and anxiety, as well as other symptom and employment measures. Some patients were considered unsuitable for the services, only received an assessment and advice, and/or were signposted to other suitable services (debt counselling, housing assistance, etc.) and not seen again. The clinical and employment outcomes reported here relate to those individuals who were seen at least twice and will have generally received at least one session of treatment (the first contact usually having focused on assessment).

### Measures

Depression was assessed with the 9-item Patient Health Questionnaire Depression Scale (PHQ-9: Kroenke, Spitzer, & Williams, 2001) which ranges from 0 to 27 with a recommended cut-off of 10 or above for distinguishing between clinical and non-clinical populations. Anxiety was assessed with the 7-item Patient Health Questionnaire Generalized Anxiety Disorder Scale (GAD-7: Spitzer, Kroenke, Williams, & Lowe, 2006) which ranges from 0 to 21. Although the latter scale was originally developed to screen for GAD, it also has satisfactory (albeit lower) sensitivity and specificity for detecting other anxiety disorders (Kroenke, Spitzer, Williams, Monahan, & Lowe, 2007). The CORE-OM (Barkham, Margison, & Leach, 2001) was given as an additional, broad-based symptom measure. Employment was assessed by a specifically developed self-report questionnaire (Department of Health, 2008b) covering type of employment (full-time, part-time, unemployed, student, retired, homemaker), receipt of statutory sick pay and state benefits.

### Procedure

It is common for studies of clinical services to have quite high levels of missing data, particularly at post-treatment. This is partly because some patients drop-out of therapy and others complete treatment before a formal assessment has been organized. In an attempt to circumvent this problem, a session-by-session outcome monitoring system that had previously been used with success in an audit of a community PTSD treatment service (Gillespie, Duffy, Hackmann, & Clark, 2002) was used. Patients were asked to complete the PHQ-9 and GAD-7 *every* session. In this way it was hoped that a measure of the severity of depression and anxiety at the last clinical contact would be available for almost everyone, including those individuals who dropped out and/or completed treatment earlier than anticipated. The CORE-OM and employment questionnaires were given less frequently (at assessment, every sixth session, and at treatment completion). In Doncaster, most of the outcome data was obtained during telephone treatment sessions with patients and entered onto the computer system by their clinician. In Newham, patients mainly completed paper versions of the questionnaires before their treatment sessions. The demonstration sites did not routinely follow-up patients after the end of treatment. However, for the purposes of this evaluation, a one-off follow-up survey was carried out. Patients who had attended at least two sessions (including the initial assessment) and had completed treatment by 1st September 2007 (i.e at the end of the service's first year) were contacted in January–February 2008 and asked to complete the main measures. All participants in the follow-up had completed treatment at least 4 months previously (mean 42 weeks).

### Statistical analysis

Clinical outcome is assessed by comparing initial assessment, post-treatment and follow-up scores on symptom measures for all patients who are considered to have started treatment (operationalized as those who attended at least two sessions, as the first session was almost always assessment) and by computing recovery rates for those individuals who at initial assessment scored above the clinical cut-off for the PHQ-9 (10 or more) and/or the GAD-7 (8 or more). Data from the last available session is used for an individual's post-treatment score. In this way, it was possible to calculate clinical outcome in almost all patients, not just those who completed treatment in a scheduled manner. To be classified as recovered, such individuals needed to score below the clinical cut-off for *both* the PHQ-9 (9 or less) *and* the GAD-7 (7 or less) at the relevant time point (post-treatment or follow-up). Pre-treatment, post-treatment and follow-up scores are compared with paired *t*-tests and repeated measures anovas. Treatment effect sizes were calculated by subtracting the post-treatment score from the initial assessment score and dividing by the pooled standard deviation.

## Results

### Data completeness

The session-by-session outcome monitoring system achieved a high level of post-treatment data completeness. Of the 1654 patients at the Doncaster site who had at least two sessions and had completed their involvement with the service by the end of the reporting period, an impressive 99.6% (1648 patients) completed a PHQ-9 and GAD-7 at post-treatment. Of the 249 patients at the Newham site who had at least two sessions and had completed their involvement with the service by the end of the reporting period, 88.3% (220 patients) completed a PHQ-9 and GAD-7 at post-treatment, a figure that is also impressive when one bears in mind that 10% of patients in Newham do not speak English or have it as their non-preferred language. At both sites, the post-treatment data completeness rates for the measures that were not given every session were substantially lower (CORE-OM: 6% in Doncaster, 56% in Newham. Employment status: 27% in Doncaster, 54% in Newham).

### Doncaster

#### Organization and staffing

The Doncaster service describes itself as a high-volume, predominantly low-intensity service, based on a stepped-care model. Proposals for such a service were being developed from 2005 onwards. The IAPT demonstration site itself went live in mid-August 2006. The central activity of the service is individual case management, largely telephone based, which offers patients guided self-help and support based on CBT principles. It is possible for patients to be referred onwards within the service to specialist CBT (termed “high-intensity therapy”) or outside the service to counselling.

The largest group of staff working in the service are 20 case managers/low-intensity workers – people with a variety of backgrounds who receive graduate or post-graduate training in primary mental health care provided by York University (one week intensive clinical skills training, then two terms of one day a week classroom-based training and one day a week practical training supervised by York University).

The case managers operate within a structured supervision framework. Supervision takes place weekly and lasts for around 1 h. It is provided by CBT therapists employed by IAPT. The decision about which patients to take to supervision is automated – it includes all those with high PHQ-9/GAD-7 scores, and also every patient after every 4th session. Case managers also have open access to specialist CBT therapists on a daily basis to discuss patient treatments and risks, as needed.

If case managers find a case needs more intensive CBT therapy, after adequate treatment duration at lower treatment steps, they can transfer the patient to one of the CBT therapists employed in the IAPT project. Normally CBT therapists spend one day a week on supervision of case managers and one day acting as the service ‘duty manager’, with their remaining days available for clinical work. The service was funded for 4 CBT therapists but had fewer in post (1.5 full-time equivalent) over the period covered by this report.

#### Referrals

##### Criteria

GPs were asked to refer to the service: “All patients with at least moderate depression (PHQ-9 of 10 or more) except those with a history of repeated treatment failure, psychotic features, personality disorder, primary drug/alcohol problems, or significant risk” and “All patients with generalized anxiety disorder (GAD), panic disorder (with or without agoraphobia), simple phobias, social phobia, and health anxiety, except those with significant suicide risk” or who “have failed to respond to at least 3 interventions”. More serious cases were to be referred to specialised secondary mental health services as were all cases of post-traumatic stress disorder and obsessive-compulsive disorder.

##### Source and demographics

Most referrals (96%) came from GPs, with 3% from other health professionals and 1% from employment agencies or self-referral. 65% of referrals were women and 99.5% were white. The age distribution was: 16% aged 18–24 years, 52% aged 25–44 years, 28% aged 45–64 years, and 3% over 65 years. The service therefore focused on working age adults.

##### Diagnoses

The service did not provide patients with a formal diagnosis. Instead, diagnoses were taken directly from GP referral forms. The guidelines given to GPs suggested using the PHQ-9 and GAD-7 to support the identification of caseness. As GPs do not usually use standardized interviews focusing on recognized diagnostic criteria (e.g. ICD-10 or DSM-IV) their diagnoses are of unknown validity. Bearing this in mind, GPs rated depression as the patients' primary problem in 95% of cases. In the remaining 5% anxiety disorders were rated as the primary problem, particularly generalized anxiety disorder (GAD: 3.9% of cases). Each other anxiety disorder was rated as the primary problem in less than 0.5% of cases. GPs considered most patients to have more than one diagnosis with GAD being the most common secondary problem (96%). It therefore appears that the Doncaster service is primarily a service for depression, generalized anxiety disorder, and mixed anxiety and depression. Other anxiety disorders that are covered by the National IAPT initiative and are common in the community (such as panic disorder, agoraphobia, and social phobia) do not appear to have been prominent in the service, while OCD and PTSD were formally excluded.

##### Initial severity

At their first meeting with the service, 82% of individuals scored above the PHQ-9, the clinical cut-off for depression (10 or higher) with 34% being considered “severe” (20 or higher). For the purposes of this report, patients are considered to be a clinical ‘case’ if they score above the clinical cut-off on the PHQ-9 *or* the GAD-7. On this criterion, 90% of the referred individuals were clinical cases.

##### Duration

Information on the duration of the presenting problems was available for around half of the referrals. Duration was judged to be less than 6 months in 33%, between 6 months and 2 years in 33% and more than 2 years in 34%. The median and mean durations of the presenting problem were 0.9 years and 2.9 years respectively.

*Medication status* at intake was recorded for 63% of individuals. Slightly over half (55%) were taking a psychotropic medication with selective serotonin re-uptake inhibitors (SSRIs) being most common (71% of all medications).

#### Treatment

The normal mode of treatment for a patient referred to the service is to be contacted immediately and first seen by a case manager about 21 days later, face-to-face, for 45 min to an hour, at a venue of their choice which is often on the GP's premises. This first session, when the patient first completes the standard questionnaires, leads to an assessment of the patient's problem (but no formal diagnosis) followed by the beginning of treatment.

The patient's beliefs, attitudes and behaviour are analysed, and a treatment plan including goals for the future are agreed. Typically a patient receives a copy of ‘A Recovery Programme for Depression’ by Karina Lovell and David Richards, while 42% are also given Chris Williams' ‘Overcoming Anxiety’. The case manager and patient will schedule a next meeting and agree on section(s) of the book for the patient to work through prior to that. At the next meeting they will reflect on that work, and agree on further work as needed.

Most subsequent sessions are held on the telephone. Patients who do not phone in at the appointed time are proactively followed-up, as agreed at the outset of treatment. Patients are also offered the alternative of face-to-face meetings. (Around 23% of subsequent meetings are face-to-face, which last on average 40 min) The average telephone session lasts 22 min. The case manager works with a computer, using an IT system designed to help manage and track patient treatment. The session begins with the case manager administering the PHQ-9 and GAD-7 over the phone. The patient answers the questions and the case manager records the answers on the computer. The majority of the session involves therapeutic engagement: discussion of the patient's current situation, reflection on progress, and agreeing a next piece of work as appropriate.

If the patient makes sufficient progress, treatment is discontinued by agreement. If the symptoms fail to improve, additional treatment options at the same intensity level are discussed and may be undertaken by the patient if appropriate; or she/he can be referred to regular face-to-face CBT from a therapist in the service (high-intensity intervention) or to a counsellor outside the service. It is also possible for a person to be referred directly to either a CBT therapist within the service (high-intensity work) or to a counsellor, when the referral is first received (i.e. without seeing a case manager). The decision is made by the service Duty Manager, but is relatively uncommon (around 6% of referrals).

#### Numbers of patients seen

**Fig. 1:**
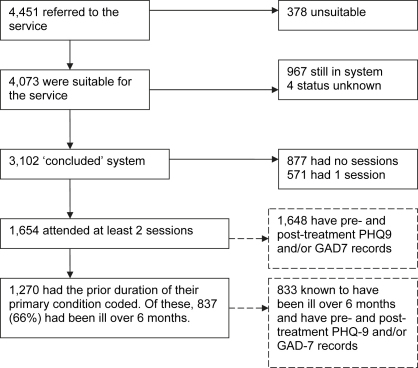
Patient progress: Doncaster.

The Doncaster site managed an impressively high number of patients. In the 13 months covered by this report (up to end September 2007), 4451 patients were referred to the programme. Fig. 1 shows what happened to them. 378 patients (8.5%) were deemed unsuitable and a further 967 patients were still in the system, either in treatment or waiting for it. This means that 3102 patients were referred, deemed potentially suitable and completed their involvement with the service in the 13 months (‘concluded cases’). Of these, 877 had no sessions. 42% of the people with no sessions did not contact the service after referral and could not be reached by the service. A further 27% refused treatment. For the remaining 31% non-attendance was mutually agreed between the service and the patient. This means that 2225 concluded cases attended one or more sessions. 571 of the 2225 came only once, and are therefore likely to have only received an assessment and brief advice. Again, they can be split into types of service conclusion. For 44%, the decision was jointly reached between the service and the patient themselves that no further treatment from IAPT was required. For many, they are likely to have been signposted to other appropriate interventions (for example, debt counselling, voluntary groups, etc). A further 22% of patients decided, independently of the service, to refuse further treatment. Finally, 34% were coded as ‘discontinued unexpectedly’; these are people who did not contact the service again after the first session and could not be reached by the service.

This leaves 1654 patients who had at least 2 sessions (including assessment). All of these patients are likely to have had at least some treatment. Our analysis of clinical outcomes focuses on this group. On average they had 4.9 sessions, comprising a total of around 2.6 h of contact (including the initial session). The median time between sessions was 12 days. The median length of treatment from first session to last was 8 weeks (the mean length is 11 weeks). Only 44 of the 1654 (i.e. 2.7%) received any sessions of face-to-face CBT from a specialist therapist (high-intensity worker). Among those who did, the mean number of CBT sessions was 5.7 (median 5.0).

The most common activity was guided self-help, using workbooks, which was done by 1442 people. 99 people did some computerised CBT. 355 people had at least one session at which none of the above took place – other common activities included providing information, medication support, and signposting to other services.

NICE. (2004a) recommendations for the stepped-care management of depression indicate that patients who fail to respond at step 2 care (low-intensity intervention here) should be offered a move to step 3 (high-intensity intervention) where face-to-face “CBT is the psychological treatment of choice” but therapists could also “consider interpersonal psychotherapy (IPT) if the patient expresses a preference for it or you think the patient may benefit from it”. Interpersonal therapy was not available in Doncaster IAPT and the number of people who stepped up to high-intensity CBT was surprisingly low. Some 650 patients were still cases (according to their PHQ-9 and GAD-7) at their last session of low-intensity work, but only 25 of them (3.8%) subsequently had high-intensity CBT within the service. A rather larger number seem to have been referred out of the service to counselling. The service referred approximately 420 patients to counselling in the period covered by the report. However, outcome data for this phase of their care is not available.

#### Clinical outcomes

Table 1 shows the initial assessment (pre) and last available session (post) questionnaire scores for patients who had at least 2 sessions (including assessment). Paired *t*-tests indicated that there were highly significant improvements on all measures: PHQ-9, *t*_(1647)_ = 47.0, *p* < .001; GAD-7, *t*_(1647)_ = 45.4, *p* < .001; CORE-OM, *t*_(91)_ = 8.9, *p* < .001. Pre to post effect sizes were large: 1.26 for the PHQ-9; 1.25 for the GAD-7; 0.98 for the CORE-OM.

If we focus on whether a person has recovered or not, 1494 of the 1,648 patients for whom we have pre- and post-treatment scores were classified as clinical cases at initial assessment. Of these, 56% had recovered (were no longer classified as cases) by the time they left the service.

##### Outcomes by duration

Further analyses investigated whether clinical outcomes (on the PHQ-9 and GAD-7) varied as a function of the duration of a patient's primary problem. Pre and post scores and recovery rates were calculated separately for patients whose problem durations were: under 3 months, 3–6 months, 6 months–1 year, 1–2 year, 2–4 years and more than 4 years. For the scores, pre to post change was not related to duration (in repeated measures anovas all assessment occasion by problem duration interactions were non-significant). However, there was a significant duration effect for recovery rates (*χ*^2^ = 13.1, *p* = .02) with the highest rates being observed when the problem duration was under 3 months (60%) or between 3 and 6 months (63%) and lowest when problem duration was over 4 years (47%).

#### Employment and benefit outcomes

Pre and post data on the employment questionnaire were available for 445 (27%) of the patients who had two or more sessions. One would not expect major changes in employment status during a short course of treatment. However, a significant number of patients who were originally off work and on Statutory Sick Pay (SSP) returned to work. This is shown in Table 2. The net increase of people at work (and not on SSP) corresponded to 4% of the treated population. This matches the assumption in the Comprehensive Spending Review cost-benefit analysis by Layard et al. (2007) that treatment raises the employment rate of those treated by 4 percentage points over the following 2 years.

#### Outcomes at follow-up

To fully evaluate the outcomes of the service, it is important to determine if the psychological and employment gains achieved at the end of treatment are largely maintained over time. As the Doncaster (and Newham) IAPT services stop collecting any information on patients once they have their last treatment session, a one-off follow-up survey was carried out for the purposes of this report in January–February 2008. Patients who had completed treatment by 1 September 2007 and had had at least two sessions were eligible for the follow-up survey. The average amount of time between the last treatment session and the follow-up survey, for the eligible group, was 42 weeks (range 16–72 weeks). The eligible group totalled 1444 people. The Doncaster site took a random sample of 893 people from this group and mailed them the PHQ-9, GAD-7, and employment questionnaire for self-completion. Individuals who did not return the questionnaires by mail were phoned and offered the chance to complete them over the phone. 452 people (51%) provided data. Respondents had a significantly lower final PHQ-9 score than the full sample, *t* = 2.03, *p* < .02.

Means (and standard deviations) for respondents PHQ-9 and GAD-7 scores are: PHQ-9; pre = 15.7 (6.3), post = 7.4 (6.9), follow-up = 8.7 (7.7). GAD-7; pre = 13.5 (5.4), post = 6.8 (6.4), follow-up = 7.6 (6.8). Repeated measures anovas indicated that patients continued to score significantly lower at follow-up than at initial assessment (*p* < .001). However, there was also a modest, but significant, increase in both PHQ-9 and GAD-7 scores between post and follow-up (*p* < .05). The recovery rate at follow-up was 50%, compared to 56% at post. Employment at follow-up showed a net increase of 10% compared to the initial assessment (343 patients provided employment data, of whom 155 were coded as employed and not claiming sick pay at initial assessment and 190 were coded the same way at follow-up).

### Newham

#### Organization and staffing

Prior to the IAPT demonstration site, Newham already had a relatively developed structure for delivering psychological therapy services, organized in three tiers. Tier 1 is a GP-practice-based, brief-therapy service, run by the Newham Primary Care Trust (PCT). Tier 2 is a PCT-wide individual, group and family therapy service (including psychodynamic, systemic and CBT therapy), run by the PCT. Tier 3 is a PCT-wide secondary-care specialist therapy service, run by the East London NHS Foundation Trust.

The Newham demonstration site consists of a new cognitive CBT service created from scratch in mid-2006 plus a linked employment service. It started in a somewhat difficult environment involving some scepticism by existing services, which made it difficult initially to obtain referrals. The service now delivers three steps of intervention, categorized according to the steps in NICE Guidance (2004a). Step 2a comprises computerised CBT, guided self-help, and group psychoeducation. Step 2b comprises brief CBT (individual and group). Step 3 comprises full CBT (individual and group). Generally, step 2a is delivered by case managers, Steps 2b and 3 by CBT therapists. The service considers step 2a ‘low-intensity’ and steps 2b and 3 as ‘high-intensity’. If patients move up from step 2b to step 3 by extending their CBT this would usually be with the same therapist but they could be allocated a different therapist if this was clinically indicated.

Development of the service occurred in two phases. In phase one, it was available to 14 GP surgeries (covering approximately one-third of the Newham working age population or around 50,000 adults), as well as to local residents referred by employers, community groups, or Jobcentre Plus. The focus was on delivering steps 2b and 3 using qualified CBT therapists. Between January and March 2007, the service entered its second phase. The referral base was broadened to include self-referrals from local residents, and to gradually incorporate all Newham GP surgeries. The focus also broadened, to include more delivery of step 2a (low-intensity) services, requiring the recruitment of more case managers. The associated employment service is provided by a voluntary organization (Mental Health Matters) and operates side-by-side with the CBT service. Employment coaches help patients to gain employment or resolve employment problems.

Clinical staffing levels in the service varied during the reporting period, rising to their highest in phase two when whole time equivalent (wte) appointments were 10.1 trained CBT therapists (including the Lead Clinician) and 6.0 case managers (low-intensity workers). 7.0 wte of these staff (3.0 wte therapists and 4.0 wte case managers) only joined in phase two.

#### Referrals

##### Criteria

Referrals of depression and all the anxiety disorders (including OCD and PTSD), as well as some other common mental health problems, were encouraged. People with a current psychosis or with a severe drug or alcohol problem which precluded them from participating fully in the therapy process, were excluded.

##### Source and demographics

Over the reporting period, 75% of referrals came from GPs and 4% came from other health professionals. The remaining 21% of patients were self-referred. However, self-referral took some time to become established and represented 42% of all referrals in the last three month period. 60% of referrals were women. 49% of all patients came from Black and Ethnic Minority (BME) groups (25% Asian, 17% Black). The age distribution was: 13% aged 18–24 years, 58% aged 25–44 years, 25% aged 45–64 years, and less than 4% under 18 or over 65 years. The service therefore focused on working age adults. 10% either did not speak, or preferred not to speak, English. They could be accommodated in the service as some staff spoke other languages and external interpreters were available.

##### Diagnoses

Staff in the Newham service provided a diagnostic assessment, based on the ICD-10 framework. Patients' primary diagnoses were as follows: 46% depression, 43% anxiety disorders and 12% other disorders. The rates of the different anxiety disorders (as a percentage of the total patient cohort) were: 3% agoraphobia, 5% social phobia, 1% other specific phobias, 6% panic disorder, 6% generalized anxiety disorder, 4% obsessive-compulsive disorder, 5% post-traumatic stress disorder, 3% health anxiety and 10% other anxiety disorder. The conditions other than depression or anxiety disorders that were considered primary problems included: anger, personality disorder, eating disorders, bipolar affective disorder, schizophrenia, and mental and behavioural disorders due to psychoactive substance use. 40% of referrals were diagnosed with two or more disorders. Grouping all disorders together (primary and secondary), the most common disorder was an anxiety disorder (45%) followed by depression (42%). From the diagnostic information, it would appear that the Newham service covered the full range of conditions relevant to the IAPT initiative.

##### Initial severity

At their first meeting with the service, 76% of individuals scored above the clinical cut-off for depression on the PHQ-9 (score of 10 or higher), with 28% being considered “severe” (20 or higher). For the purposes of this report, patients are considered to be a clinical ‘case’ if they score above the clinical cut-off on the PHQ-9 *or* the GAD-7. On this criterion, 86% of the referred individuals were clinical cases of depression and/or anxiety disorders.

##### Duration

Entry to the service was restricted by previous duration of the patient's condition: either the condition must have been in place for six months or more; or for three months together with substantial negative impact on accommodation, employment or associated physical health. Information on the duration of the presenting problems was recorded for 59% of the referrals. Duration was judged to be less than 6 months in 22%, between 6 months and 2 years in 17% and more than 2 years in 61%. The median and mean durations of the presenting problem were 3.3 years and 7.0 years respectively, indicating a relatively chronic population.

##### Medication status

At intake *at least* 20% of patients were taking psychotropic medication (see Clark et al., 2008 for further details), with SSRIs being most common (73% of all medications).

#### Treatment

All people referred initially attend an assessment with a qualified therapist or an assistant therapist working with the supervision of a qualified therapist. The suggested treatment allocation depends on the condition and its severity. The intention is that if low-intensity (step 2a) treatments (guided self-help, computerised CBT, group psychoeducation) could be useful, the patient is started on these. Escalation up the stepped grades of treatment occurs if the patient has not improved after 4 h of step 2a or 8 h of step 2b. If at 20 h within the service (including all levels of treatment) a patient still has not improved, they will usually be referred to secondary care (e.g. the community mental health team). Some patients could be allocated directly to high-intensity CBT within IAPT, for example if low-intensity treatments are known to be ineffective (such as for patients suffering post-traumatic stress disorder), or if there is substantial risk to self or others. In practice, staffing limitations meant many step 2a treatments only began to be delivered during phase two of the service delivery, from mid-March 2007. This initially limited the implementation of the treatment allocation system outlined above. The extent of this limitation is evident by comparing the patients who had completed their treatment within the period of this report and patients who had at least two sessions but were still in the service at the end of the reporting period. 25% of the former but 66% of the latter had experienced a low-intensity intervention.

The normal mode of treatment is for a patient referred to the service to be contacted within a few days and first seen for assessment about 14 days later. Assessment usually lasts around an hour. For some, ‘flexible engagement’ occurs before formal assessment. This involves discussion between the patient and service staff about their illness and the nature of the service. This is intended to encourage referrals to take up the service. Around 29% of referrals have at least one ‘flexible engagement’ contact. The average duration of a flexible engagement contact is 9 min, and almost all (86%) on the telephone.

Following assessment, the patient is allocated to a treatment. For guided self-help two workbooks are used, ‘Overcoming Anxiety: A Five Areas Approach’, and ‘Overcoming Depression: A Five Areas Approach’, both by Chris Williams. Computerised CBT is also available but interestingly was rarely taken up by patients. For high-intensity therapy, there is a lead therapist for each disorder (panic/phobia, OCD, PTSD, etc).

Both the CBT and employment services are based at the newly established Newham Psychological Treatment Centre. Service users can choose whether they would like therapy and employment services to take place at the Centre, at their GP surgery, elsewhere, or over the phone. Excluding flexible engagement and assessment, the majority of contacts are done face-to-face (84% face-to-face versus 16% on the phone); of the face-to-face contacts, 68% take place at the Centre, 21% take place at the GP surgery, and 11% at other locations. The average length of a face-to-face session is 47 min, and of a telephone session is 18 min.

#### Numbers of patients seen

**Fig. 2:**
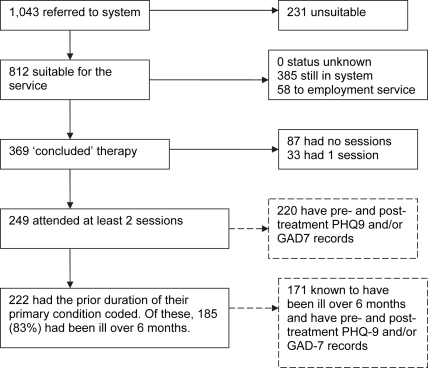
Patient progress: Newham.

In the 13 months covered by this report (up to end September 2007), 1043 patients were referred to the Newham IAPT programme. Fig. 2 shows what happened to them. 231 were found unsuitable, 58 were referred direct to the employment service and a further 385 patients were still in the system, either in treatment or waiting for it. This means that 369 patients were referred, deemed potentially suitable and completed their involvement with the service in the 13 months (‘concluded cases’). Of these, 87 had no sessions and 33 had only one session. This means that 249 concluded cases attended at least 2 sessions (including the assessment). All of these are likely to have had at least some treatment. Our analysis of clinical outcomes focuses on this group. On average they had 8.2 sessions, comprising a total of around 7.2 h of contact (including initial assessment session). The median time between sessions is 14 days. The median length of treatment from first session to last is 16 weeks (the mean is 18 weeks).

The most common activity was receiving step 3 (high-intensity) CBT from a specialist therapist. This applied to 183 of the 249 (74%). Among these patients, the mean number of CBT sessions was 7.3 (median 7.0). Other activities included low-intensity ‘step 2a’ interventions, such as guided self-help, group psychosocial education, and CCBT. 42 of the 249 (17%) had at least one session of low-intensity work. The most common was guided self-help (22 individuals). 7 individuals had computerised CBT. 36 individuals also received support from the employment service. Within a stepped-care system, patients who begin with a low-intensity intervention and fail to recover should be offered a high-intensity intervention. Of the 22 patients who were still cases at their last low-intensity session, 32% subsequently had high-intensity CBT within the service. Most of the remainder were recorded as having dropped out of treatment.

#### Clinical outcomes

Table 3 shows the initial assessment (pre) and last available session (post) questionnaire scores for patients who had at least 2 sessions (including assessment). Paired *t*-tests indicated that there were highly significant improvements on all measures: PHQ-9, *t*_(219)_ = 13.2, *p* < .001; GAD-7, *t*_(220)_ = 15.7, *p* < .001; CORE-OM, *t*_(140)_ = 11.9, *p* < .001. Pre to post effect sizes were large: 1.06 for the PHQ-9; 1.26 for the GAD-7; 1.19 for the CORE-OM.

If we focus on whether a person has recovered or not, 197 of the 220 patients for whom we have pre- and post-treatment scores were classified as clinical cases at initial assessment. Of these, 55% had recovered (were no longer classified as cases) by the time they left the system.

##### Outcomes by duration

Further analyses investigated whether clinical outcomes (on the PHQ-9 and GAD-7) varied as a function of the duration of a patient's primary problem. Pre and post scores and recovery rates were calculated separately for patients whose problem durations were: under 6 months, 6 months–1 year, 1–2 year, 2–4 years and more than 4 years. There were no significant duration effects.

##### Outcomes by ethnicity

Newham is an ethnically mixed PCT. Of the patients who received at least two sessions and had concluded their involvement with the service, 54% (134 people) were white and 46% came from BME groups (27% asian, 13% black, 5% other groups). In order to determine whether ethnicity was related to outcome, change in scores from pre to post-treatment and recovery rates were compared between the groups. There were no significant effects, indicating that individuals from different ethnic groups were equally likely to benefit from the service, once they have gained access to treatment. Recovery rates were: white 50%; Asian 66%, Black 54%, Other 50%.

#### Employment and benefit outcomes

Pre and post data on the employment questionnaire were available for 135 (54%) of the patients who had two or more sessions. Table 4 shows the data. At post-treatment a net increase of 10% in the number of people in work and not receiving Statutory Sick Pay (SSP) was observed. The increase comes mainly from reducing the number of people on Statutory Sick Pay (a decrease of 6%) and a decrease in numbers in the “other” category (not employed, and not receiving benefits or SSP) of 4%.

#### Outcomes at follow-up

The Newham IAPT service stopped collecting information on patients once they had their last treatment session. In order to obtain a picture of the longer-term outcome of patients seen in the service, a one-off follow-up survey was carried out in January–February 2008. Patients who had completed treatment by 1 September 2007 and had had at least two sessions were eligible for the follow-up survey. The average amount of time between last treatment session and the follow-up survey, for the eligible group, was 42 weeks (range 17–74 weeks). The eligible group totalled 165 people. The service mailed to them the PHQ-9, GHQ-7, and employment questionnaire for self-completion. Individuals who did not return the questionnaires by mail were phoned and offered the chance to complete them over the phone. 60 people (36%) provided data. Respondents did not differ from the total sample in terms of their pre or post PHQ-9 or GAD-7 scores or the duration of their problems.

Means (and standard deviations) for respondents PHQ-9 and GAD-7 scores are: PHQ-9; pre = 15.1 (6.6), post = 7.4 (6.4), follow-up = 8.5 (6.8). GAD-7; pre = 13.8 (5.3), post = 6.5 (5.2), follow-up = 7.8 (5.9). Repeated measures anovas indicated that patients continued to score significantly lower at follow-up than at initial assessment (*p* < .001). The modest increase in PHQ-9 and GAD-7 scores between post and follow-up was not significant (*p* > .16). The recovery rate at follow-up was 42%, compared to 57% at post.

#### Comparison between self-referrals and GP referrals

Newham encouraged self-referral as an experiment to see if this route into the service facilitates access for groups who are not well served by GP referral alone. When self-referral cases were compared with those from the GP, they did not differ in the initial severity of their depression (PHQ-9) or anxiety (GAD-7). The prior duration of their presenting problem was longer (7.5 years mean versus 6.9; medians are 4.0 years versus 3.0), but the difference was not significant. There was a significant difference in ethnicity between the two referral routes with individuals from the black community being more prominent among self-referrals than among GP referrals (self-referrals: *n* = 203 of whom 22.2% were black; GP referrals: *n* = 688 of whom 15.9% were black: *χ*^2^ = 4.17, *p* = .041). There was also a significant difference in the relative commonness of different diagnoses, with both social phobia and obsessive-compulsive disorder being more common among self-referrers than GPs referrals (Social phobia: 13% of self-referrals versus 3% of GP referrals, *p* < .001. Obsessive-compulsive disorder: 8% of self-referrals versus 3% of GP referrals, *p* < .05). Finally, the outcomes of patients referred by the two routes were compared using referral type (self vs GP) by time (pre vs post) repeated measures anovas. The referral type by time interactions were not significant for either the PHQ-9 or the GAD-7, suggesting the self-referrers and GP referrals do not differ in outcome. However, the latter conclusion needs to be viewed as preliminary as the late adoption of self-referral means that the number of self-referrers who had completed treatment within the period of this report is small. The relevant means and (standard deviations) are: PHQ-9 self-referrers, *n* = 21, pre mean = 13.1 (8.1) post mean = 7.9 (6.3); gp referrals, *n* = 195, pre mean = 15.6 (6.0), post mean = 8.2 (7.2); interaction *F* = 1.29, *p* = .26. GAD-7 self-referrers, *n* = 21, pre mean = 12.4 (6.4) post mean = 6.5 (5.5); gp referrals, *n* = 195, pre mean = 13.9 (5.0), post mean = 6.8 (5.9); interaction *F* = 0.64, *p* = .42.

Taken together the above comparisons between self-referrers and GP referrals suggest that opening up a service to self-referral is beneficial because it improves access for some underrepresented groups (individuals from the black community and those with two of the common anxiety disorders that are not always picked-up by GPs) and does not seem to attract cases that are any less severe than those normally referred by GPs. Encouragingly, the outcome date that is so far available suggests that once they access the service, self-referrers are likely to have clinical outcomes that are as good as GP referrals.

### Why data completeness matters

**Fig. 3:**
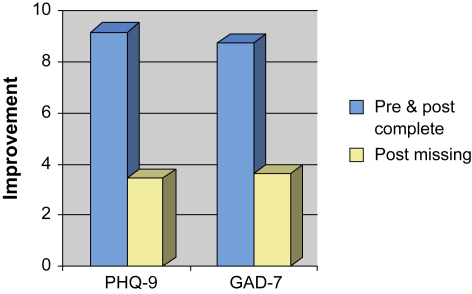
Improvement in PHQ-9 and GAD-7 scores between initial assessment (pre) and last available session (post) in people who either completed the CORE-OM at pre and post or who failed to complete the CORE-OM at post. Data from the Newham Demonstration site.

Missing data, particularly at post-treatment, is common in evaluations of the outcomes achieved in routine clinical services. There is controversy about the importance of such missing data (Clark, Fairburn, & Wessely, 2007). Is it reasonable to assume that the clinical outcomes of patients for whom post-treatment data is missing will be as good as those for patients with complete data or is it possible that patients with missing data are likely to have done less well (or better) overall? The Doncaster and Newham data allow us to address this issue empirically. Both sites used two outcome monitoring systems. One was the session-by-session system (using the PHQ-9 & GAD-7) which achieved almost complete pre to post data (99% for Doncaster, 88% in Newham). The other was a more conventional, less frequently sampled, outcome monitoring system that was used with another symptom measure (CORE-OM) and the employment questionnaire. The latter system was associated with much lower pre–post treatment data completeness. To determine whether missing data matters, we compared the pre- and post-treatment scores on the PHQ-9 and GAD-7 in those individuals who did, or did not, provide pre–post treatment data for the CORE-OM or the employment questionnaire. Separate repeated measures anovas were performed on the PHQ-9 and GAD-7 data from each site. For each analysis, the repeated measures factor (time) was pre vs post scores on the PHQ-9 or GAD-7. The between subjects factor (“data completeness”) was whether participants did or did not have complete pre-post data on the CORE-OM or the employment questionnaire. For all but one of the analyses, there was a significant time by data completeness interaction (all *p* < .001). Inspection of the means indicated that in both sites patients who had complete data on the CORE-OM or the employment questionnaire improved more on their anxiety scores (GAD-7) than patients who had incomplete data. The same pattern was observed for depression (PHQ-9) scores. In Newham, patients who had complete data on the CORE-OM or the employment questionnaire showed greater improvement in depression. In Doncaster, patients who had complete data on the employment questionnaire, but not CORE-OM, showed significantly greater improvement in depression. To obtain an estimate of the extent to which patients with complete data on the conventional outcome monitoring system improved more that those with incomplete data, separate pre-post effects sizes were computed for the two groups. The effect sizes on the PHQ and GAD were on average 1.72 times greater for the data complete group (range 1.09–2.47). Fig. 3 illustrates the data completeness effect.

Why might individuals who fail to provide post-treatment data on the conventional outcome monitoring system fare less well in therapy? One possible explanation is that they have had less sessions of therapy, partly because they are more likely to drop-out. Consistent with this suggestion, in both sites patients who completed pre and post versions of either the CORE-OM or the employment questionnaire had significantly more therapy sessions before leaving the service (all *p* < .001).

Taken together, the data completeness analyses strongly suggest that patients who fail to provide post-treatment data in conventional outcome monitoring systems are likely to have done less well clinically than the patients who provide post-treatment data. As a consequence, it seems likely that services run the risk of overestimating their effectiveness if they fail to collect outcome data on almost all the people they treat, including those who drop-out or otherwise terminate after only a few sessions.

## Discussion

Both demonstration sites had substantial achievements over their first thirteen months, against a background of considerable difficulties. These difficulties included an uncertain and delayed beginning to funding and no assurance of long-term funding in order to facilitate staff recruitment. The fact that they achieved as much as they did is a testament to the outstanding dedication and hard work of those involved.

Both demonstration sites were new start-ups. The Doncaster site had existed in conception for about 12 months before it started. It hit the ground running and achieved a truly impressive level of throughput from the start. The Newham site had a standing start and initially it had serious organisational difficulties in obtaining sufficient referrals. Its throughput eventually increased but was still not as high as it should be, given its staffing. It underwent service redesign, putting more focus on step 2a (low-intensity) services in phase two. It is anticipated that the increased emphasis on low-intensity interventions will increase throughput in the future, as it has done in Doncaster.

The clinical populations served by the sites are very different. The Doncaster site focused predominantly on individuals in whom depression was considered by GPs to be the main problem, whereas Newham focused on depression and the full range of anxiety disorders. Individuals seen in Doncaster are predominantly white, whereas Newham has an ethnically mixed population with a significant number of individuals who do not speak English. Finally, a larger proportion of individuals seen in Newham had their problem for more than 2 years (34% in Doncaster, 61% in Newham).

In what follows we summarize the main achievements of the two sites, discuss key issues of interpretation and outline some issues for the future development of IAPT services for depression and the anxiety disorders.

### Achievements

#### Numbers treated

An impressive number of people were assessed and treated by the demonstration sites. During the thirteen months covered by this report (August 2006–September 2007) nearly 5500 people were referred to the two sites, of whom around 4800 were considered suitable for the services. Approximately 3500 of these individuals concluded their involvement with the services during the period of this report, with the remainder still in the system. Of the concluded cases, around 1900 received at least two sessions (including the assessment interview) with most having pre- and post-treatment scores on standardized outcome measures. The numbers seen in Doncaster are particularly impressive and highlight the importance of low-intensity work (guided self-help, computerised CBT etc) for achieving high patient throughput with those disorders for which a stepped-care model is appropriate.

#### Psychological benefits

In terms of therapeutic results, both demonstration sites achieved good recovery rates (55–56%) for people who had at least some treatment (i.e. attended two or more sessions). These recovery rates need to be considered in the context of what we know about natural recovery in depression and the anxiety disorders and also the clinical outcomes that have been reported in randomized controlled trials of CBT.

A substantial literature shows that some patients with depression and/or anxiety disorders recover without major professional help. It therefore seems inevitable that some of the patients who received treatment in Doncaster or Newham would have improved even without access to the services. The most sensitive way of determining whether a service has an added effect that enhances recovery in a substantial number of people is a randomized controlled trial in which some people receive treatment in the service immediately and some do so after a delay (the “wait list control condition”). Differences in recovery between people who receive immediate treatment compared to those on the wait list show the added benefit of treatment. As this evaluation is an observational, prospective cohort study, there is no wait list control condition. In the absence of such a condition, one has to rely on benchmarking to other samples in order to decide whether treatment was effective. This is a less sensitive method than the randomized controlled trial. If the improvements one observes with treatment are much larger than those observed in benchmark samples of people who had no, or minimal, treatment, one can be fairly confident that treatment was effective. However, if the improvements observed with treatment are within the range of the benchmarks it is difficult to draw clear conclusions. There could be a small beneficial effect of treatment but this cannot be established without precise data on natural recovery within the particular population under investigation.

The literature suggests that natural recovery varies with the prior duration of a clinical disorder. Several studies (Catalan, Gath, Edmonds, & Ennis, 1984; Kendrick et al., 2006; Spijker et al., 2002; Tennant, Hurry, & Bebbington, 1981) have looked at recovery in recent onset cases of depression and/or anxiety in primary care and have reported recovery rates of 50–70% over the next few months in patients who received modest GP “treatment as usual” that excluded formal psychological therapy. In contrast, studies that have recruited cases with a prior duration of 6 months or over tend to report very low recovery rates in wait list samples. For example, in Posternak and Miller's (2001) meta-analysis of wait list control groups the average recovery rate from depression was approximately 20%. In randomized controlled trials of CBT for anxiety disorders that have focussed exclusively on patients with a duration of more than 6 months (Clark et al., 1994, 1998, 2006; Ehlers, Clark, Hackmann, McManus, & Fennell, 2005) recovery rates rarely exceed 5% in the wait list.

In both demonstration sites, the majority of patients (66% in Doncaster, 83% in Newham) reported having been depressed or anxious for more than 6 months. The recovery rates in these patients (52% for both sites) comfortably exceed the 5–20% one might expect from natural recovery or minimal intervention. It therefore seems reasonable to conclude that the treatment offered in the IAPT demonstration sites is frequently effective in these cases^1^. The position is less clear for the minority of cases with a more recent onset. Here the recovery rates fall within the range of those that have been reported in control samples so one cannot be confident that the treatment lead to improvements over and above those that might have happened in any case. Such beneficial effects may be present but they would need to be demonstrated in a randomized controlled trial.

As benchmarking suggests that treatment has a beneficial effect in the majority of cases, we should now ask whether the improvements that were observed with treatment are more or less in line with what one might expect from randomized controlled trials of CBT? It is difficult to make precise comparisons between trials and clinical services because the two differ in numerous respects. For example, patients in trials tend to be heavily selected (there are numerous exclusion criteria) and more highly motivated. However, the IAPT programme drew on the trials literature to set itself the target that 50% of people who are treated in IAPT will “move towards recovery” (see www.iapt.nhs.uk). The recovery rates observed in Doncaster and Newham with patients who have had at least some treatment are in line with this target.

#### Employment effects

The effects of the IAPT services on employment are also encouraging. At post-treatment, the observed increase in employment without claiming sick pay was of 4% in Doncaster and 10% in Newham, giving an overall figure of 5% for the total cohort of people treated in Doncaster and Newham. This is supportive of the assumptions made in the cost-benefit analyses that supported the initial case for the IAPT initiative.

#### Self-referral

Ever since the creation of the UK National Health Service in 1948, GPs have acted as a “gate keeper” to specialist treatment services. Self-referral opportunities have been rare. However, concern that a GP only access system may disadvantage some individuals with mental health problems led the Newham Demonstration site to experiment with self-referral. Comparisons between self-referrers and GP referrals supported the idea that self-referral may be particularly helpful for promoting access to treatment for some community groups or clinical conditions that tend to be underrepresented in GP referrals. As a consequence, the UK Government approved the use of self-referral in the national roll-out of IAPT services that is currently in progress (see IAPT Implementation Plan: National Guidelines for regional delivery, available at www.iapt.nhs.uk).

#### Outcome monitoring

Concern about the sometimes high levels of missing data at post-treatment in evaluations of routine clinical services led Doncaster and Newham to adopt a session-by-session outcome monitoring system. In this way, it was hoped that a post-treatment (last available session) assessment would be available for almost everyone who received treatment. The session-by-session monitoring was successful with both demonstration sites achieving very high completeness levels for their pre- and post-treatment assessments. This means that one can be confident that the improvements in depression (PHQ-9) and anxiety (GAD-7) reported here accurately reflect the overall impact of the services. An interesting feature of the outcome monitoring at both sites was the parallel use of a session-by-session system (for the PHQ-9 and GAD-7) and a more conventional pre and post only system (for the CORE-OM and employment questionnaire). The conventional, pre and post only, system was associated with much lower levels of data completeness and there was good evidence that patients who fail to provide post-treatment data in this system tend to have improved less in terms of their depression and anxiety. The clear benefits of the session-by-session system have lead to it being incorporated into the data collection plans for the national IAPT roll-out (see IAPT Outcomes Toolkit 2008/09, available from www.iapt.nhs.uk).

#### Ethnicity

The UK has an ethnically diverse population. It is important that the new IAPT services meet the needs of each of its ethnic groups. The national IAPT programme is carefully monitoring ethnicity to ensure that different groups have equal access to the services. In this respect, it is encouraging that the demonstration site that has the most ethnically diverse community: 1) succeeded in engaging White, Black and Asian groups, especially through the use of self-referral, and 2) found that these groups did not differ in their recovery rates, once they had accessed treatment in the service.

#### Successful dissemination

Taken together, the findings from Doncaster and Newham in terms of numbers of people treated, clinical and employment outcomes, referral routes and ethnicity show that treatment protocols that have been developed in research studies can be applied with reasonable success in varied clinical settings with a large number of people from diverse backgrounds.

### Some issues for the future development of IAPT services

#### Compliance with NICE guidelines

The two demonstration sites were works in progress. Despite their impressive achievements, neither could be described as comprehensive services that implemented the NICE guidelines for the psychological treatment of depression and all the anxiety disorders. Newham covered the full range of problems but its early emphasis on high-intensity treatment meant that it initially failed to reach the desired scale. Doncaster focused more exclusively on those problems for which a stepped-care model is particularly indicated and placed a very strong emphasis on low-intensity work (such as guided self-help). This produced a high throughput but step up to high-intensity CBT rarely occurred even in individuals who continued to meet caseness criteria at the end of low-intensity work. Ideally, low and high-intensity interventions should be widely available and described to patients in a manner that ensures that, if individuals have failed to respond to a low-intensity intervention, they are still motivated to try a high-intensity intervention, if appropriate. Ways of ensuring this happens consistently in the National IAPT roll-out need to be explored.

NICE guidance varies between the different disorders covered by the IAPT initiative. In order to establish which guidance is relevant, a provisional diagnosis needs to be established for each patient on entry to the service. How this can be done reliably without greatly extending assessment time, particularly with low-intensity workers, also needs to be considered.

#### Clinical follow-up

Depression is a recurring condition. Psychological treatments are particularly interesting as they bring with them the potential to achieve enduring change. However, neither demonstration site routinely followed-up patients to determine whether their gains had been maintained. We would recommend that in the future IAPT sites consider including a routine follow-up 3–6 months after treatment completion, with the addition of a few booster sessions at that stage if there are signs of deterioration. The follow-up specially conducted for this report encouragingly showed that at 4–12 month follow-up most of the psychological gains achieved during treatment were maintained. However, there was a modest amount of drop back that could perhaps have been reduced by a planned clinical follow-up and routinely including established relapse prevention procedures in the treatment programmes.

## Figures and Tables

**Table 1:** Initial assessment (pre) and last available session (post) scores for patients who had at least two sessions (including assessment) and had completed their involvement with the Doncaster IAPT service in the reporting period.

		Pre	Post
PHQ-9	Mean (SD)	15.8 (6.2)	7.5 (6.9)
	N	1648	1648
GAD-7	Mean (SD)	13.9 (5.2)	6.8 (6.2)
	N	1648	1648
CORE-OM	Mean (SD)	1.88 (0.59)	1.18 (0.82)
	N	92	92

**Table 2:** Changes in employment from initial assessment (pre) to last available session (post) for patients who had at least two sessions (including assessment) and had completed their involvement with the Doncaster IAPT service in the reporting period.

		Pre-treatment				
		Benefit recipient (IB, IS, JSA)	Receiving Statutory Sick Pay	Employed (no SSP)	Other	Total
Post-treatment	Benefit recipient (IB, IS, JSA)	115	6	8	15	144
	Receiving Statutory Sick Pay	3	27	13	0	43
	Employed (no SSP)	9	27	149	7	192
	Other	14	3	5	44	66

	Total	141	63	175	66	445

**Table 3:** Initial assessment (pre) and last available session (post) scores for patients who had at least two sessions (including assessment) and had completed their involvement with the Newham IAPT service in the reporting period.

		Pre	Post
PHQ-9	Mean (SD)	15.3 (6.2)	8.2 (7.2)
	N	221	221
GAD-7	Mean (SD)	13.7 (5.1)	6.8 (5.8)
	N	221	221
CORE-OM	Mean (SD)	1.83 (0.61)	1.07 (0.67)
	N	140	140

**Table 4:** Changes in employment from initial assessment (pre) to last available session (post) for patients who had at least two sessions (including assessment) and had completed their involvement with the Newham IAPT service in the reporting period.

		Pre-treatment				
		Benefit recipient (IB, IS, JSA)	Receiving Statutory Sick Pay	Employed (no SSP)	Other	Total
Post-treatment	Benefit recipient (IB, IS, JSA)	38	0	1	5	44
	Receiving Statutory Sick Pay	0	2	0	0	2
	Employed (no SSP)	2	6	54	8	70
	Other	3	2	2	12	19

	Total	43	10	57	25	135

## Footnotes

1 Around half of the patients who received psychological treatment in Doncaster and at least 20% of those in Newham were also taking psychotropic medication. While it seems likely that this may have contributed to recovery in such cases, there were no significant differences in recovery rates between those taking, or not taking, medication (59% vs 52% in Doncaster).
